# DNAJB3/HSP-40 cochaperone improves insulin signaling and enhances glucose uptake *in vitro* through JNK repression

**DOI:** 10.1038/srep14448

**Published:** 2015-09-24

**Authors:** Mohamed Abu-Farha, Preethi Cherian, Irina Al-Khairi, Ali Tiss, Abdelkrim Khadir, Sina Kavalakatt, Samia Warsame, Mohammed Dehbi, Kazem Behbehani, Jehad Abubaker

**Affiliations:** 1 Biochemistry and Molecular Biology unit, Dasman Diabetes Institute, Kuwait; 2 Diabetes Research Center, Qatar Biomedical Research Institute, Education City, Doha, Qatar

## Abstract

Heat shock response (HSR) is an essential host-defense mechanism that is dysregulated in obesity-induced insulin resistance and type 2 diabetes (T2D). Our recent data demonstrated that DNAJB3 was downregulated in obese human subjects and showed negative correlation with inflammatory markers. Nevertheless, DNAJB3 expression pattern in diabetic subjects and its mode of action are not yet known. In this study, we showed reduction in DNAJB3 transcript and protein levels in PBMC and subcutaneous adipose tissue of obese T2D compared to obese non-diabetic subjects. Overexpression of DNAJB3 in HEK293 and 3T3-L1 cells reduced JNK, IRS-1 Ser-307 phosphorylation and enhanced Tyr-612 phosphorylation suggesting an improvement in IRS-1 signaling. Furthermore, DNAJB3 mediated the PI3K/AKT pathway activation through increasing AKT and AS160 phosphorylation. AS160 mediates the mobilization of GLUT4 transporter to the cell membrane and thereby improves glucose uptake. Using pre-adipocytes cells we showed that DNAJB3 overexpression caused a significant increase in the glucose uptake, possibly through its phosphorylation of AS160. In summary, our results shed the light on the possible role of DNAJB3 in improving insulin sensitivity and glucose uptake through JNK repression and suggest that DNAJB3 could be a potential target for therapeutic treatment of obesity-induced insulin resistance.

Diabetes mellitus is a chronic metabolic disorder caused by defects in insulin secretion and/or insulin action^1^,^2^,^3^,^4^. The resulting chronic hyperglycemia is associated with numerous complications such as retinopathy, neuropathy and nephropathy. Over 90% of cases of diabetes mellitus are of type 2 diabetes (T2D) a form of diabetes characterized by increased insulin demand caused by insulin resistance^2^,^5^. Obesity induced insulin resistance has been well recognized as a main cause of T2D^3^,^4^,^6^. Obesity is characterized by a chronic, low-grade inflammatory response in key metabolic tissues^2^,^3^,^5^,^7^. The uncontrolled inflammatory reaction and alteration of the stress response system play an important role in the inhibition of insulin receptor signaling cascade causing disruption of systemic metabolic homeostasis^3^,^4^,^8^,^9^,^10^.

One of the key stress response systems that are dysregulated in obesity-induced insulin resistance and T2D is the heat shock response (HSR) which is a crucial host-defense mechanism against stressful conditions^11^,^12^,^13^. Patients with T2D have reduced expression of heat shock proteins (HSPs). HSPs are chaperone proteins that play major role in mediating protein refolding, tissue protection, tissue repair, and cellular homeostasis^14^,^15^. For example, HSP27 binds and inhibits the stress kinase IKKβ^16^,^17^ and regulates TNF-α induced NF-κB activation^17^. Activation of certain HSPs such as HSP72 has been shown to be protective against obesity induced insulin resistance through its ability to tightly-bind JNK and prevents its phosphorylation^11^,^18^,^19^,^20^. JNK has been implicated in the mechanism of obesity-induced insulin resistance; germ-line ablation of JNK prevents both diet-induced obesity and insulin resistance 21,22,23. Taken together, these data highlight the importance of the HSPs cellular response in mitigating damages associated with obesity-mediated insulin resistance.

Our recent data demonstrated that DNAJB3, an HSP-40 protein family member, is downregulated in response to obesity in peripheral blood mononuclear cells (PBMCs) and subcutaneous adipose tissue and showed negative correlation with key pro-inflammatory markers such as IP-10 and RANTES^24^. Physical exercise was able to restore the expression of DNAJB3 in obese subjects with a concomitant decrease of phosphorylated JNK. Our results also showed that DNAJB3 protein expression was reduced following the activation of the Endoplasmic Reticulum (ER) stress following treatments with ER-stress inducers such as tunicamycin and palmitate^24^. Furthermore, DNAJB3 was also shown to co-immunoprecipiate with JNK and IKKβ stress kinases along with HSP72 and thus, suggesting its potential role in modulating their activities^24^. However, DNAJB3 expression pattern in diabetic subject and its mode of action are yet to be discovered.

In this study, we investigated the expression level of DNAJB3 and its mode of action in relation to JNK and insulin signaling in obese non-diabetic and obese T2D Subjects. Our result showed reduction in the expression of DNAJB3 gene and protein in PBMC and subcutaneous adipose tissue of obese T2D subjects compared to obese non-diabetic subjects. We also showed for the first time that DNAJB3 overexpression improved insulin signaling and glucose uptake in pre-adipocytes. On the other hand, JNK inhibition caused by DNAJB3 overexpression resulted in the activation of IRS-1 and subsequently AKT, AS160 phosphorylation highlighting a possible mechanism through which DNAJB3 could be improving glucose uptake.

## Materials and Methods

### Study population

The study was conducted on adult obese T2D (BMI = 30–40 kg/m^2^) and obese non-diabetic subjects. Informed written consents were obtained from all subjects before their participation in the study, which was approved by the Ethical Review Board of Dasman Diabetes Institute and carried out in line with the guidelines of the ethical declaration of Helsinki. Participants falling under any of the following categories; morbid obese subjects (i.e. BMI >40 kg/m^2^) and participants with prior major illness, were excluded from the study as previously reported^24^,^25^.

### Blood and tissue sampling

Venous peripheral blood and subcutaneous adipose tissue biopsies were obtained from obese non-diabetics and T2D subjects and processed as reported previously (24). In brief, PBMCs were prepared from blood using Ficoll-Hypaque density gradient centrifugation method. Plasma and serum were prepared using vacutainer tubes, aliquoted and stored at −80 °C. Subcutaneous superficial adipose tissue biopsies (about 1 g) were obtained from the periumbilical area by surgical biopsy after a local anesthesia. Once removed, the biopsy was rinsed in cold PBS, divided into 4 pieces and stored at −80 °C until assayed.

### Measurement of gene expression by Real-time Quantitative PCR

Total RNA was extracted from frozen adipose tissue and PBMCs using RNeasy Lipid Tissue Mini Kit and AllPrep RNA/Protein Kit, respectively (Qiagen, Inc., Valencia, CA). Total RNA was isolated from PBMC and adipose tissue biopsies of obese non-diabetic (n = 8) and obese-diabetic (n = 8). The cDNA was prepared from total RNA sample using High Capacity cDNA Reverse Transcription Kits (Applied Biosystems, Foster City, CA). qRT-PCR was performed on Rotor-Disc 100 system using SYBR Green normalized to *Gapdh* (Qiagen, Inc., Valencia, CA). PCR primers used were: *DNAJB3* For., 5′-ATCCGAGGCCATCAAGAAG-3′; *DNAJB3* Rev., 5′-CCACCTGCTTGAATCTCCTC-3′; *Gapdh* For., 5′-AACTTTGGCATTGTGGAAGG-3′ and *Gapdh* Rev., 5′-TGTGAGGGAGATGCTCAGTG-3′. Relative expression was assessed by using the ΔΔCT method^26^.

### Cell Culture, plasmids and transfection

Human embryonic kidney (HEK-293), 3T3-L1 cell lines were obtained from American Type Culture Collection (Rockville, Baltimore, MD). Cells were cultured in Eagle’s Minimum Essential Medium (EMEM) supplemented with 10% fetal bovine serum and penicillin/streptomycin. Human DNAJB3 gene cloned in pCMV6 backbone vector and c-terminally tagged with Myc-DDK tags was purchased from Origene (OriGene Technologies, Inc., Rockville, MD). Empty pCMV6 vector with Myc- and DDK- tags was used as a control in all transfections. Lipofectamine® LTX was used to transfect HEK-293 and 3T3-L1 cells. Briefly, 95% confluent cells were transfected with 24 *μg* of DNA according to the manufacturer protocol (Invitrogen, Carlsbad, CA). Following transfection, HEK-293 cells were incubated in complete media for 48 hours. Cells were treated overnight with palmitate after 24 hrs of transfection with either DNAJB3 or the pCMV6 empty plasmid. Palmitate was purchased as Palmitic acid (Sigma Aldrich, St. Louis, MO) and prepared in fatty acid free and low endotoxin Bovine Serum Albumin (BSA) (Sigma Aldrich, St. Louis, MO) at a final concentration of 125 μM. 3T3-L1 cells were harvested after 96 hours of transfection for the glucose uptake experiment. DNAJB3 specific siRNA were purchased from Origene (OriGene Technologies, Inc., Rockville, MD). Three different siRNA molecules were tested compared to scrambled siRNA at different concentrations (0.1, 1, 5 and 10 nM). A final concentration of 10 nM of DNAJB3 specific and scrambled siRNA was used for transfection in HEK-293 cells using Lipofectamine 3000. Following transfection, HEK-293 cells were incubated in complete media for 48 hours. Cells were treated overnight with 125 μM palmitate after 24 hrs of transfection.

### Co-immunoprecipitations

Approximately 2 × 10^7^ of HEK-293 transfected cells were harvested after 48 hours of transfection and washed twice with ice-cold PBS. Cells were then lysed in 1 ml of modified RIPA lysis buffer (50 mM Tris-HCl, pH 7.4, 150 mM NaCl, 1 mM EDTA and 1% Triton X-100) supplemented with mini complete protease inhibitor cocktail (Roche Diagnostics, Laval, Quebec) for 30 min at 4 °C. Extracts were centrifuged at 14,000 rpm for 10 minutes at 4 °C to remove cell debris. 500 μg of total cell lysates were added to 100 μl of 50% slurry of anti-FLAG M2 affinity agarose beads (Sigma Aldrich, St. Louis, MO), pre-equilibrated with ice cold washing buffer (50 mM Tris-HCl pH 7.4 and 150 mM NaCl) and incubated overnight at 4 °C with continuous end-over-end rotation^24^. Protein complexes were collected by centrifugation and washed four times in washing buffer and bound proteins were eluted with 100 μl of 3 × FLAG tag peptide at 150 μg/ml as recommended by the manufacturer (Sigma Aldrich, St. Louis, MO). Specific antibodies against DNAJB3 and JNK were then used to check for their interactions by immunoblotting.

### Western blot analysis

Western blots were carried out on whole PBMC extracts or cell extracts prepared in RIPA buffer (50 mM Tris-HCl pH7.5, 150 mM NaCl, 1% Triton ×100, 1 mM EDTA, 0.5% Sodium deoxycholate and 0.1% SDS). Total proteins were extracted from PBMC of obese non-diabetic (n = 4) and obese-diabetic (n = 4) participants and analyzed by western blotting using the indicated antibodies. Protein concentration was determined by Bradford method using globulin as a standard and 20 μg of proteins were resolved on 10% SDS-PAGE gels. Proteins were then transferred onto PVDF membranes, blocked with 5% non-fat dried milk in Tris-buffered saline containing 0.05% Tween 20 (TBST) for 1 h at room temperature (RT) and then probed with the primary antibody for overnight at 4 °C. After washing, the membranes were incubated with horseradish peroxidase-conjugated secondary antibody for 2 h at RT and finally, protein bands were visualized by chemiluminescence and the images were captured using the Versadoc 5000 system (BioRad, Hercules, CA). The primary antibodies used in this study are raised against DNAJB3 (Proteintech Group, Inc., Chicago, IL), p-JNK detects the level of phosphorylated p46 and p54 SAPK/JNK, p-AKT, p-AS160 and their total protein antibodies were purchased from Cell Signaling (Cell Signaling Technology, Inc., Danvers, MA). Both p-IRS-1 antibodies were purchased from Abcam (Abcam Company, Burlingame, CA). Actin (Santa Cruz Biotechnology, Santa Cruz, CA) and GAPDH (Millipore, Temecula, CA) were used as internal controls. For densitometric analysis, the intensity of the bands was determined using Quantity One Software (BioRad, Hercules, CA).

### Immunohistochemistry

Formalin fixed, paraffin embedded adipose tissue samples were prepared and used to make sections for immunohistochemical studies as described previously^24^. Briefly, sections were deparaffinized and the antigens were retrieved at high-temperature using antigen unmasking solution (Dako, Denmark). The endogenous peroxidase was quenched using 3% H_2_O_2_ (Merck Schuchardt, Gemany) for 60 min at RT. Sections were blocked with 5% fat-free milk for 60 min at RT followed by 1% BSA for another 60 min and then, incubated at 4 °C for overnight with primary antibodies as mentioned in the Figures. After washing, sections were stained with horseradish conjugated secondary antibody (Dako, Denmark) for 60 minutes at RT. Colors were developed using DAB kit (Dako, Denmark) and sections were counterstained with hematoxylin (Sigma Aldrich, St. Louis, MO). Quantification of the immunohistochemical staining data was done using Aperio software version 6.3 (Molecular Devices, Downingtown, PA) with an established arbitrary threshold.

### Glucose (2-NBDG) uptake experiment in HEK-293 cell line

HEK-293 cells transfected with DNAJB3 and pCMV6 empty vector were plated at 1 × 10^5^ cells per well in a 96-well plate. After 24 h of incubation (~70% confluence), the culture medium was removed from each well and replaced with 100 μl of Glucose free culture medium to starve the cells. After the cells are starved for an hour in glucose free media, the media is replaced with culture media supplemented with Fluorescent tagged D-glucose analog (2-NBDG, 150 μg/ml) and Insulin (0.1 μM) and incubated at 37 °C for 1 hr. After 1 hr, cells were washed with provided wash buffer and the fluorescence was read at wavelength 485/535 nm as instructed by the manufacturer (Cayman, Ann Arbor, MI).

### Differentiation of 3T3-L1 and glucose uptake

3T3-L1 cells were differentiated as described previously^27^. In brief, 3T3-L1 cells were cultured using basic medium (DMEM supplemented with 10% bovine calf serum and 100 units/mL penicillinstreptomycin) at 37 °C in a 5% CO_2_ incubator. After reaching 100% confluence (day 1), the cells were initiated for the adipocyte differentiation by incubating with induction medium (basic medium supplemented with 0.5 mmol/L isobutylmethylxanthine, 0.25 mmol/L dexamethasone, and 10 mmol/L insulin). Two days after induction (day 3), the medium was changed to basic medium supplemented with insulin only for an additional 1 day and seeded into 96 well plates. On day 4, Glucose uptake experiment was done as indicated above. On day 4, 3T3-L1 cells with or without DNAJB3 were processed for oil red O (ORO) staining for confirming adipocytes differentiation.

### Statistical analysis

Student’s t-test was used to determine significance of difference in means between the two groups as indicated in the figure legends. Correlations between variables were calculated with the Spearman’s rank correlation test. Differences were considered statistically significant at *P*-values less than 0.05.

## Results

### Reduced expression of the *DNAJB3* gene in obese-T2D subjects in PBMC and adipose tissue

**Figure 1:**
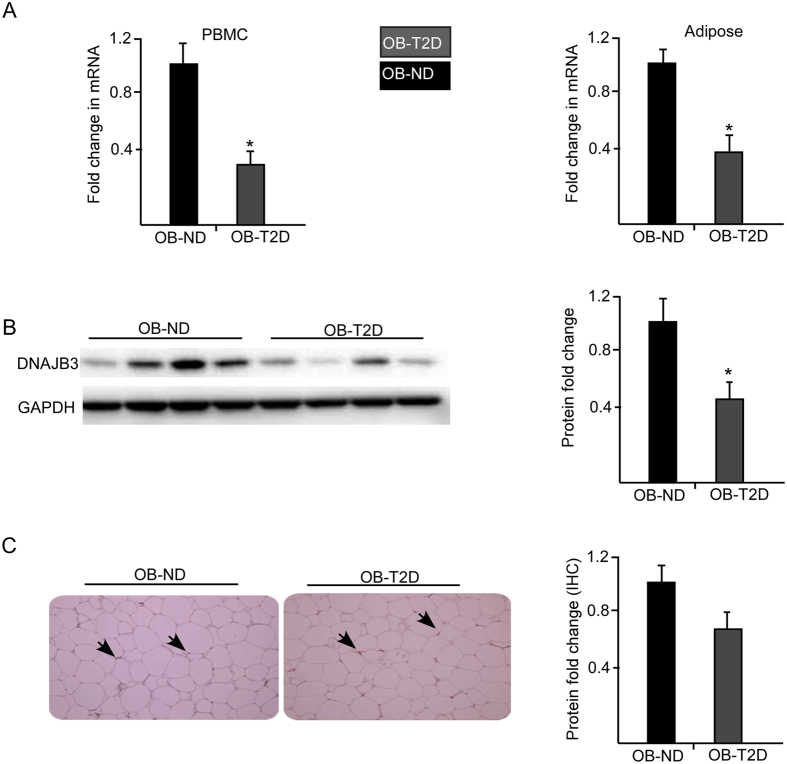
Downregulation of *DNAJB3* gene in obese diabetic subjects. (**A**) Total RNA was isolated from PBMC and adipose tissue biopsies of obese non-diabetic (n = 8) and obese-diabetic (n = 8) and subjected to quantitative analysis using real-time PCR. The data are presented as fold changes in obese-diabetic compared to obese non-diabetic subjects. (**B**) Total proteins were extracted from PBMC of obese non-diabetic (n = 4) and obese-diabetic (n = 4) participants and analyzed by western blotting using the indicated antibodies. The bands were quantified as described in materials and methods and the relative intensity was determined after correction with GAPDH that was used as an internal control to ensure equal loading. The data are presented in the form of graphs on right as fold changes compared to obese non-diabetic group. (**C**) IHC staining using fat adipose tissue biopsies obese non-diabetic (n = 8) and obese-diabetic (n = 8) participants. Aperio software was used to quantify positive staining (indicated by arrows) and the values are illustrated at the bottom as fold changes compared to lean.*P < 0.05 as determined using student’s t-test.

Our earlier report has shown a significant reduction in *DNAJB3* transcripts from PBMC and adipose tissue of obese non-diabetics subjects compared to the lean controls (24). We further looked at the expression level of *DNAJB3* to include obese-T2D subjects. We performed real-time PCR analysis using RNA isolated from obese non-diabetics and obese-T2D subjects (n = 8 for each group). Quantitative RT-PCR analysis for RNA isolated from PBMCs and adipose tissues revealed a 2.5 to 3.3-fold reduction in DNAJB3 expression in obese-T2D subjects, compared to obese non-diabetics, (P = 0.035 and 0.02 respectively, Fig. 1A). These results indicate that T2D is associated with a significant reduction in the expression of the *DNAJB3* gene in the studied tissues.

### Validation of the gene expression data by Western Blotting and Immunohisto-chemistry

Protein expression level of DNAJB3 gene was measured by immuno blotting in PBMCs and immunohistochemical (IHC) analysis in adipose tissue from selected obese non-diabetics and obese-T2D subjects. As shown in Fig. 1B, protein expression analysis showed a significant reduction in the expression of DNAJB3 protein in obese-T2D subjects (*P* = 0.03) compared to obese non-diabetic. Consistent with the western blot analysis, IHC analysis using subcutaneous adipose tissue biopsies isolated from obese non-diabetics and T2D (n = 8 each) subjects showed similar pattern of reduction in DNAJB3 expression as seen in PBMCs (Fig. 1C). Hence, both PBMCs and subcutaneous adipose tissues showed reduction in DNAJB3 level in agreement with the gene expression data for *DNAJB3*.

### DNAJB3 binding to JNK stress kinase

**Figure 2:**
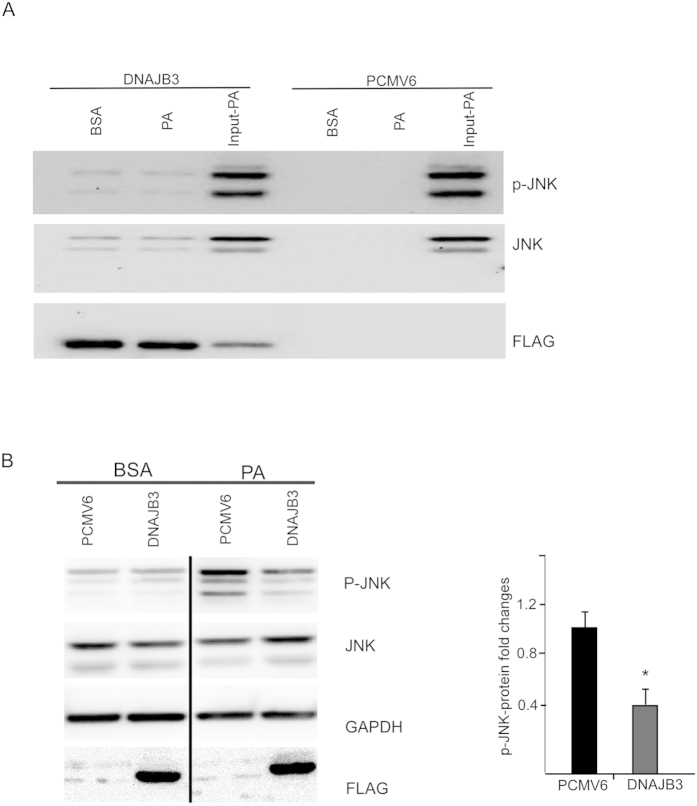
DNAJB3 binds to JNK stress kinase *in vitro* and reduce its activity. (**A**) HEK-293 cells transfected with Flag-tagged DNAJB3 and empty vector were treated overnight with palmitate (PA) and proteins lysates were co-immunprecipitated with anti-Flag antibody. Eluted proteins were subjected to western blot analysis using JNK antibody. Non-treated (BSA) and PCMV6 empty vector were run in parallel and used as controls. Anti-Flag antibody was also used to monitor transfection efficiency and binding of the recombinant proteins to the anti-Flag antibody. Total cell lysate used for the co-immunoprecipitation were run and labeled as input to show equal loading in both treatments. (**B**) HEK-293 cells transfected with Flag-tagged DNAJB3 and empty vector were treated overnight with 125 *μM* palmitate and whole cell lysate proteins were prepared from HEK-293 cells and separated on SDS-PAGE and subjected to Western blotting using the JNK, p-JNK, and FLAG-DNAJB3 and GAPDH antibodies as indicated. Relative quantification of the phosphorylated JNK was normalized to GAPDH and to its corresponding total protein. Extra lanes have been cropped as indicated by the black line; however, gels have been run under the same experimental conditions. *P < 0.05 as determined using student’s t-test, N = 3.

JNK is a pro-inflammatory molecule that has been linked to the mechanism of obesity-induced insulin resistance^21^,^22^,^23^. Our earlier data has shown an *in vivo* inverse correlation between the levels of DNAJB3 and activated JNK and the binding of DNAJB3 to JNK^24^. Therefore, we decided to check the level of binding between DNAJB3 and JNK upon stimulation with palmitate using HEK-293 cell line. As shown in Fig. 2, we were able to detect the presence of JNK bands in the co-immunoprecipitated protein complex prepared from cells transfected with DNAJB3 clone. Under the same conditions, these bands were not detected in lysates prepared from cells transfected with the empty vector control and thus, demonstrating the specificity of the interactions. However, palmitate treatment did not seem to cause any change in the level of binding between DNAJB3 and JNK (Fig. 2A). On the other hand, DNAJB3 was interacting with p-JNK but there was no change in the level of p-JNK under both palmitate and BSA treatments.

### DNAJB3 overexpression in HEK-293 cell line prevented free palmitate-induced JNK phosphorylation and impaired insulin signaling

**Figure 3:**
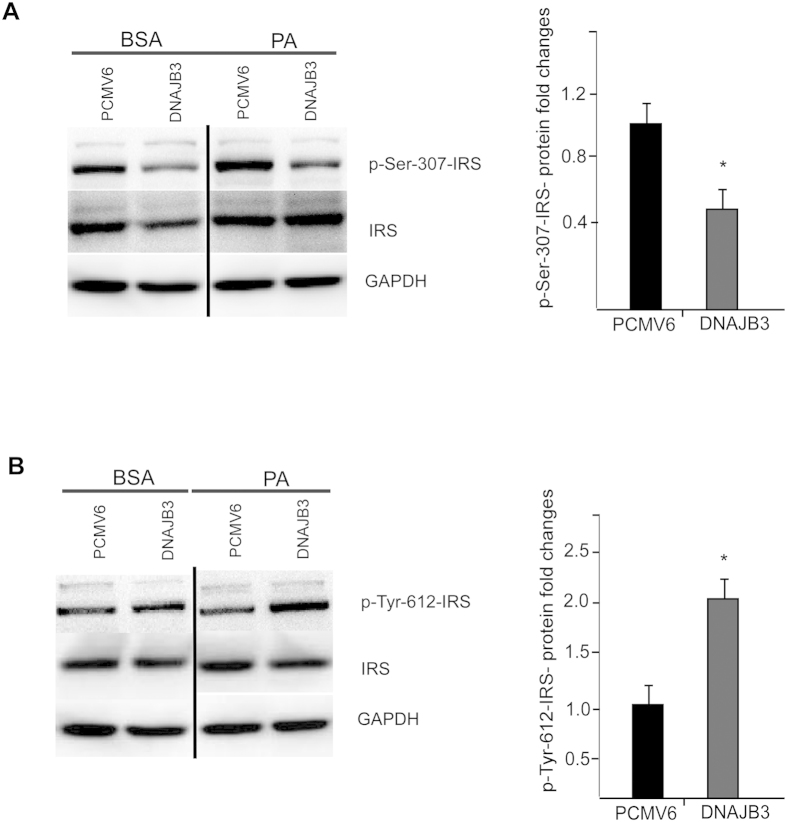
DNAJB3 overexpression stimulates IRS-1 activity. Representative immunoblots and quantification of p-IRS-1 Ser-307 (Fig. 3A) and Tyr-612 positions (Fig. 3B) in HEK-293 cells transfected with Flag-tagged DNAJB3 and empty vector following treatment overnight with 125 *μM* palmitate. Relative quantification of the p-IRS-1 serine and tyrosine positions were normalized to GAPDH and to their corresponding total proteins. Extra lanes have been cropped as indicated by the black line; however, gels have been run under the same experimental conditions. *P < 0.05 as determined using student’s t-test, N = 3.

**Figure 4:**
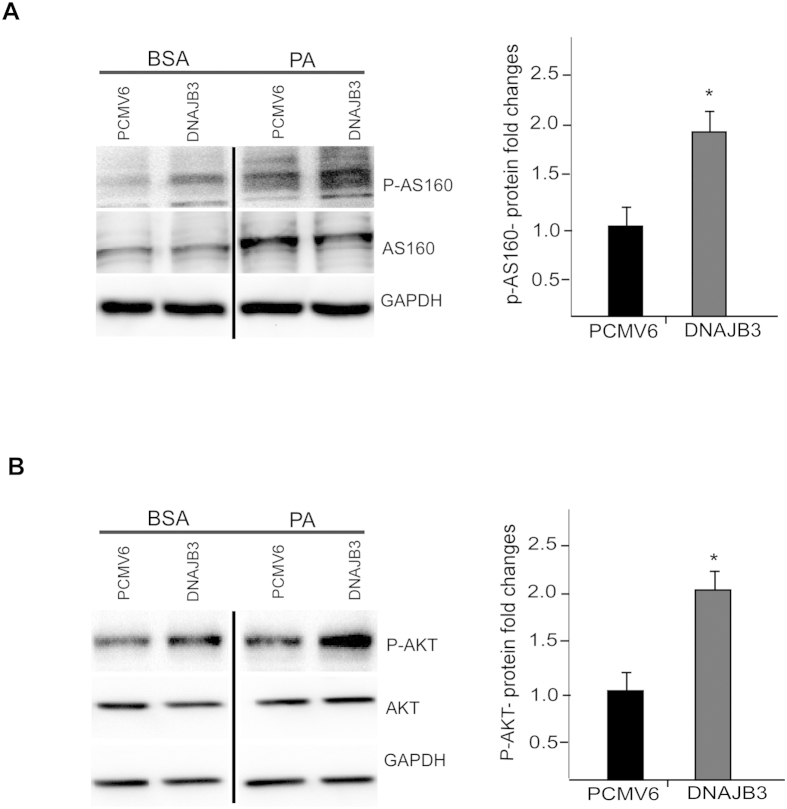
DNAJB3 overexpression mediates AKT and AS160 phosphorylation. Representative immunoblots and quantification of phosphorylated AKT (Fig. 4A) and AS160 proteins (Fig. 4B) in HEK293 cells transfected with Flag-tagged DNAJB3 and empty vector were treated overnight with 125 *μM* palmitate. Relative quantification of the phosphorylated AKT and AS160 were normalized to GAPDH and to their corresponding total proteins. Extra lanes have been cropped as indicated by the black line; however, gels have been run under the same experimental conditions. *P < 0.05 as determined using student’s t- test, N = 3.

JNK stress kinase is a major modulator of insulin singling pathway through the phosphorylation of IRS-1 on Ser-307, rendering it a poor substrate for the activated insulin receptor. Since our co-immunoprecipitation analysis has shown a binding between DNAJB3 and JNK stress kinase, we decided to investigate a possible involvement of DNJAB3 in modulating JNK activity. Under palmitate treatment, overexpression of DNAJB3 caused a significant reduction in JNK expression compared to the empty vector (representative blot in Fig. 2B). No changes were detected in total JNK expression (Fig. 2B). Consequently, the reductions in JNK activity has led to the reduction of the IRS-1 phosphorylation at the Ser-307 residue (Fig. 3A) and increase of IRS-1 phosphorylation at the Tyr-612 residue that is usually associated with improvement in insulin sensitivity (Fig. 3B). In order to assess the improvement in insulin sensitivity, we explored downstream targets that usually associate with insulin level such as AKT and AS160. Our result showed a two fold increase in the phosphorylation of AKT and AS160 in the DNAJB3 transfected cells when compared to the control cells (Fig. 4A,B). On the other hand, down regulation of DNAJB3 using siRNA showed slight increase in JNK activation and reduction in insulin signaling related proteins such as AS160 and AKT and p-Tyr-612 IRS1 (supplementary Figure 1). Taken together, these results support the possible DNAJB3 role in improving insulin sensitivity through the inhibition of JNK stress kinase.

### DNAJB3 Overexpression increased the glucose uptake in HEK-293 and 3T3-L1 cell lines and improved JNK and insulin signaling

**Figure 5:**
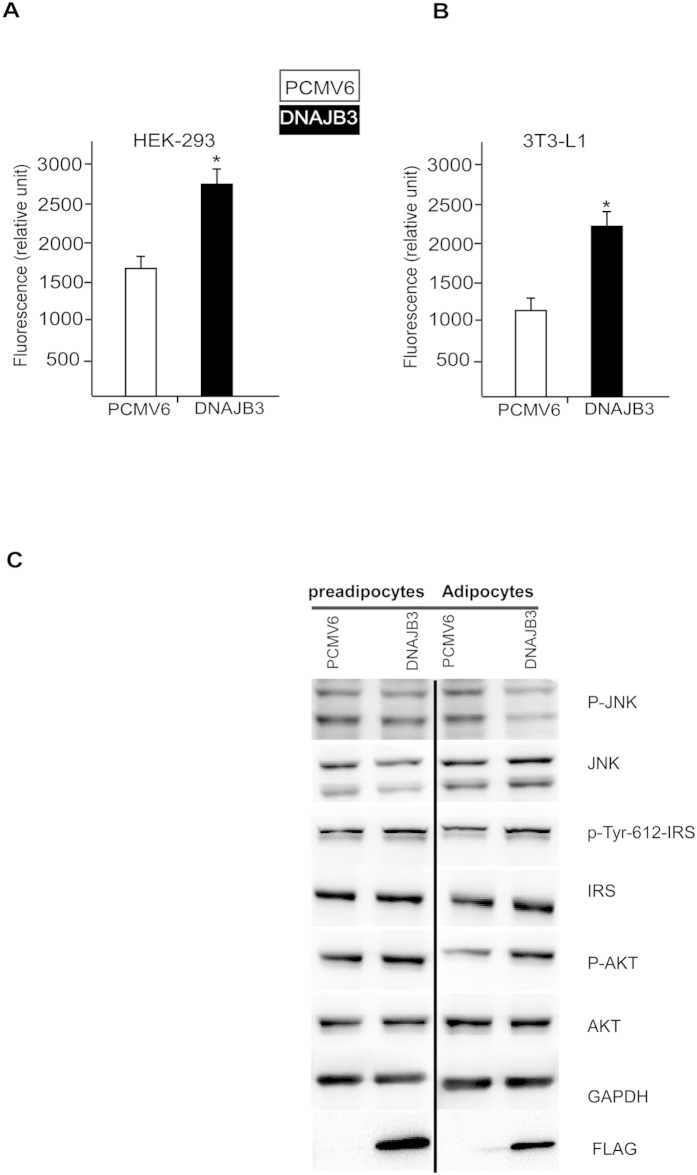
DNAJB3 overexpression increases the glucose uptake in HEK-293 and AS160 phosphorylations. (**A**) Glucose uptake analysis was done on HEK-293 cells transfected with Flag-tagged DNAJB3 and empty vector. (**B**) Glucose uptake analysis was done on differentiated 3T3-L1 pre-adipocyte cell line at day 4. At the day of the glucose uptake experiment, the cells were starved for an hour in glucose free media. After that, the media was replaced with culture media supplemented with Fluorescent tagged D-glucose analog (2-NBDG, 150 μg/ml) and Insulin (0.1 μM) and incubated at 37 °C for 1 hr. After 1 hr, cells were washed with provided wash buffer and the fluorescence was read at wavelength 485/535 nm as instructed by the manufacturer (Cayman, Ann Arbor, MI). Significant increase in glucose uptake was observed in both cell lines that express DNAJB3 compared to the control. This experiment was repeated three times and similar results were obtained. *P < 0.05 as determined using student’s t-test, N = 3. (**C**) The effect of DNAJB3 overexpression on JNK and insulin signaling pathway in 3T3-L1 in pre-adipocytes and after differentiation into adipocytes. Whole cell lysate were prepared from 3T3-L1 cells and separated on SDS-PAGE and subjected to Western blotting using the JNK, p-JNK, p-Ser-307 IRS1 and p-Tyr612 IRS1, IRS1, p-AKT, AKT and GAPDH antibodies as depicted in the figure. Extra lanes have been cropped as indicated by the black line; however, gels have been run under the same experimental conditions.

**Figure 6:**
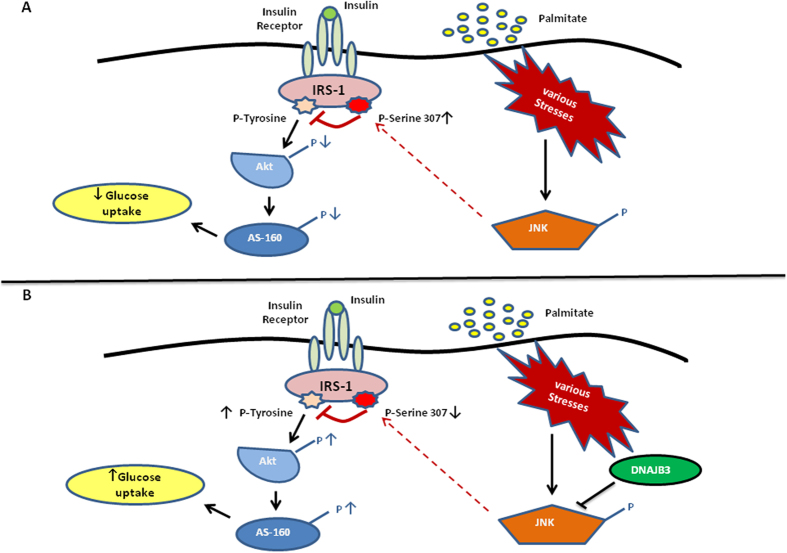
A proposed model for DNAJB3 mode of action and its involvement in insulin signaling and glucose uptake.

AS160 phosphorylation is important in the mobilization process of GLUT4 transporter to the cell membrane and the improvement of the glucose uptake. Since our DNAJB3 *in vitro* data has shown that DNAJB3 increases AS160 phosphorylation, we decided to assess DNAJB3 role in glucose uptake. HEK-293 and 3T3-L1 cells were transfected with either DNAJB3 or empty vector control. Over 60% increase in glucose uptake was observed in HEK-293 cells expressing DNAJB3 compared to the control (Fig. 5A). Similar and more pronounced glucose uptake (>2-fold increase) result was observed in 3T3-L1 pre-adipocytes cells transfected with DNAJB3 in comparison to the control (Fig. 5B). Furthermore, we showed that the overexpression of DNAJB3 in differentiated 3T3-L1 cells resulted in reducing p-JNK and improving insulin signaling related proteins such as AKT, and IRS1 as shown in (Fig. 5C). The significant increase in glucose uptake observed supports the role of DNAJB3 in improving insulin signaling (Fig. 6). This experiment was repeated three times and similar results were obtained.

## Discussion

There is a widespread clinical interest in the protective role of HSPs against a variety of diseases, including obesity, insulin resistance and diabetes^8^,^28^,^29^,^30^. Attenuation of this important host defense system is associated with various clinical manifestations and pathological disorders such as diabetes^11^,^14^,^19^,^28^. Our earlier data has demonstrated the down-regulation of DNAJB3 expression in obese non-diabetic subjects and suggested a potential protective role for DNAJB3 in obesity^24^. The current study is showing a reduction in DNAJB3 gene and protein expression in T2D obese subjects compared to non-diabetic obese. It also confirms the binding between DNAJB3 protein and JNK, shedding further light on the role of DNAJB3 in JNK suppression. Finally, *in vitro* expression of DNAJB3 protein caused a significant improvement in the insulin signaling pathway as shown by the increase in phosphorylation of IRS-1 (tyrosin), AKT and AS160, which eventually improved the glucose uptake in two different cell lines supporting the potential role of DNAJB3 in improving insulin signaling and glucose uptake in obesity and insulin resistance.

The low expression of DNAJB3 in obese T2D subjects compared to non-diabetic subjects suggests a possible role of DNAJB3 reduction in the progression to diabetes. The state of low HSPs that prevails in diabetes promotes the inflammatory response by increasing the expression of pro-inflammatory cytokines, and activation of pro-inflammatory kinases such as JNK and IKK^17^,^20^. Increased inflammation, in turn, promotes inhibitory phosphorylation of IRS-1 which inhibits insulin signaling in insulin sensitive tissues. Thus, a vicious cycle is established, which hastens the progression of diabetes. The reduction of DNAJB3 expression resembles the expression behavior of some of the inducible HSPs (HSP72 and HSP25) that inhibit JNK and IKKβ stress kinases, and are often low in diabetic cases^17^,^20^. These findings provide additional evidence on the importance of DNAJB3 and other components of the HSR to protect against metabolic disorders associated with obesity.

Beside their chaperone activity, HSPs are well known for their anti-inflammatory and anti-stress properties by binding to JNK and IKKβ stress kinases and concomitantly suppressing their activities^11^,^12^,^18^,^31^,^32^,^33^. Under our experimental conditions, we identified the stress kinase JNK as binding partner for DNAJB3 protein (Fig. 2A). Interestingly, palmitate treatment did not influence the binding between the two proteins suggesting that the mechanism by which DNAJB3 can impair JNK activity is not likely to be via degradation of JNK but rather a different mechanism.

To further elucidate the effect of DNAJB3 on metabolic diseases and to develop a model for its mode of action, we conducted a series of *in vitro* cell line experiments using palmitate as a JNK activator. JNK pathway can be activated by a variety of cellular stresses that include fatty acids, free radicals, heat shock, ischemia, osmotic shock, and pro-inflammatory cytokines such as TNF-α and IL-1β^21^. In this study, transfected HEK-293 cell line with DNAJB3 has shown a significant reduction in JNK phosphorylation as compared to the control. Since our binding assay under palmitate treatment did not seem to affect the activity of JNK, it is possible to assume that DNAJB3 might function as an inhibitor of JNK by preventing its activation through upstream kinases such as MEK kinase 1 (MEKK1) or its equivalent MAPK kinase kinases as seen previously with HSP72^34^,^35^. Earlier studies have suggested several mechanisms for JNK inactivation by HSP72^20^,^36^. Park *et al.*, have proposed that HSP72, through binding to JNK, may prevent JNK activation by a JNK kinase such as SEK1 or may inhibit the enzymatic activity of JNK as well as the interaction between JNK and its substrate, c-JUN^20^. Yaglom *et al.*, have suggested that the down-regulation of JNK by HSP72 is likely to involve regulation of upstream phosphatases such as duel leucine zipper-bearing kinase (DLK)^36^. On the other hand, increasing DNAJB3 level reduces the activity of JNK and probably lessens inflammation since JNK activation has been known to associate with inflammation. Waetzig *et al.*, have shown that JNK inhibition can reduce the LPS-induced metabolic activity and inflammatory reactions such as induction of the AP-1 target genes cyclooxygenase-2 (Cox-2), TNF-alpha, monocyte chemoattractant protein-1 (MCP-1), and interleukin-6 (IL-6) in response to LPS^37^.

Increasing evidence points at chronic tissue oxidative stress as one of the underlying factors in the etiology of insulin resistance^38^. The link between oxidative stress and insulin resistance is the stress activated kinases which can phosphorylate IRS-1 on serine residues^39^,^40^,^41^. DNAJB3 inhibition of JNK pathway pointed to a potential interaction between DNAJB3 and IRS-1 since several kinases have been implicated in serine phosphorylation of IRS-1 including JNK^39^, IKKβ^41^ and mammalian target of rapamycin (mTOR)^42^. In our study, overexpression of DNAJB3 has resulted in the reduction of p-Ser-307-IRS-1 and increased tyrosine phosphorylation of IRS-1 possibly leading to an improvement in IRS-1 signaling (Fig. 3). IRS-1 plays a pivotal point in the switch to insulin resistance, where serine phosphorylation of IRS-1 causes a conformational change in the IRS-1, preventing insulin induced tyrosine phosphorylation^22^.

Furthermore, the phosphorylation and activation of IRS-1 on tyrosine position by insulin eventually leads to AKT and AS160 activation^9^,^42^. AKT is a serine kinase located downstream of IRS-1/PI3K in insulin signaling pathway. Overexpression of DNAJB3 has improved IRS-1 signaling by tyrosine phosphorylation of IRS-1 which subsequently mediated the PI3K/AKT pathway activation hence increasing AKT phosphorylation. Likewise, *in vitro* DNAJB3 overexpression has resulted in the increase of the phosphorylation of AS160 protein. AS160 is a Rab GTPase-activating protein that mediates the mobilization of GLUT4 transporter protein to the cell membrane and thereby improves glucose uptake^43^. Taken together, our results show the important role that DNAJB3 might play in improving insulin sensitivity and glucose uptake. In line with the protein expression data, DNAJB3 overexpression in differentiated 3T3-L1 pre-adipocyte and HEK-293 has caused a significant increase in glucose uptake, which further supports the possible role of DNAJB3 in improving insulin sensitivity and glucose uptake. Based on the present data, we suggest a model for DNAJB3 mode of action with reference to its role in insulin signaling and glucose uptake focusing on the significant reduction of JNK phosphorylation and increase in IRS-1, AKT and AS160 activities (Fig. 6).

In summary, our data provides compelling evidence that DNAJB3 can modulate insulin signaling pathway by improving insulin acquisition and glucose uptake. This improvement was tightly associated with the prevention of JNK phosphorylation and increase in AKT and AS160 phosphorylation. This suggests that DNAJB3 could be a potential target for therapeutic treatment of obesity-induced insulin resistance.

## Additional Information

**How to cite this article**: Abu-Farha, M. *et al.* DNAJB3/HSP-40 cochaperone improves insulin signaling and enhances glucose uptake *in vitro* through JNK repression. *Sci. Rep.* **5**, 14448; doi: 10.1038/srep14448 (2015).

## Supplementary Material

Supplementary Information
