# Supplementary material for: DNAJB3/HSP-40 cochaperone improves insulin signaling and enhances glucose uptake in vitro through JNK repression
Source: Sci Rep. 2015 Sep 24;5:14448. doi: 10.1038/srep14448 (PMC4585859; doi:10.1038/srep14448)
Supplement: Supplementary Information [file srep14448-s1.pdf]

DNAJB3/HSP-40 cochaperone improves insulin signaling and enhances glucose uptake *in vitro* through JNK repression

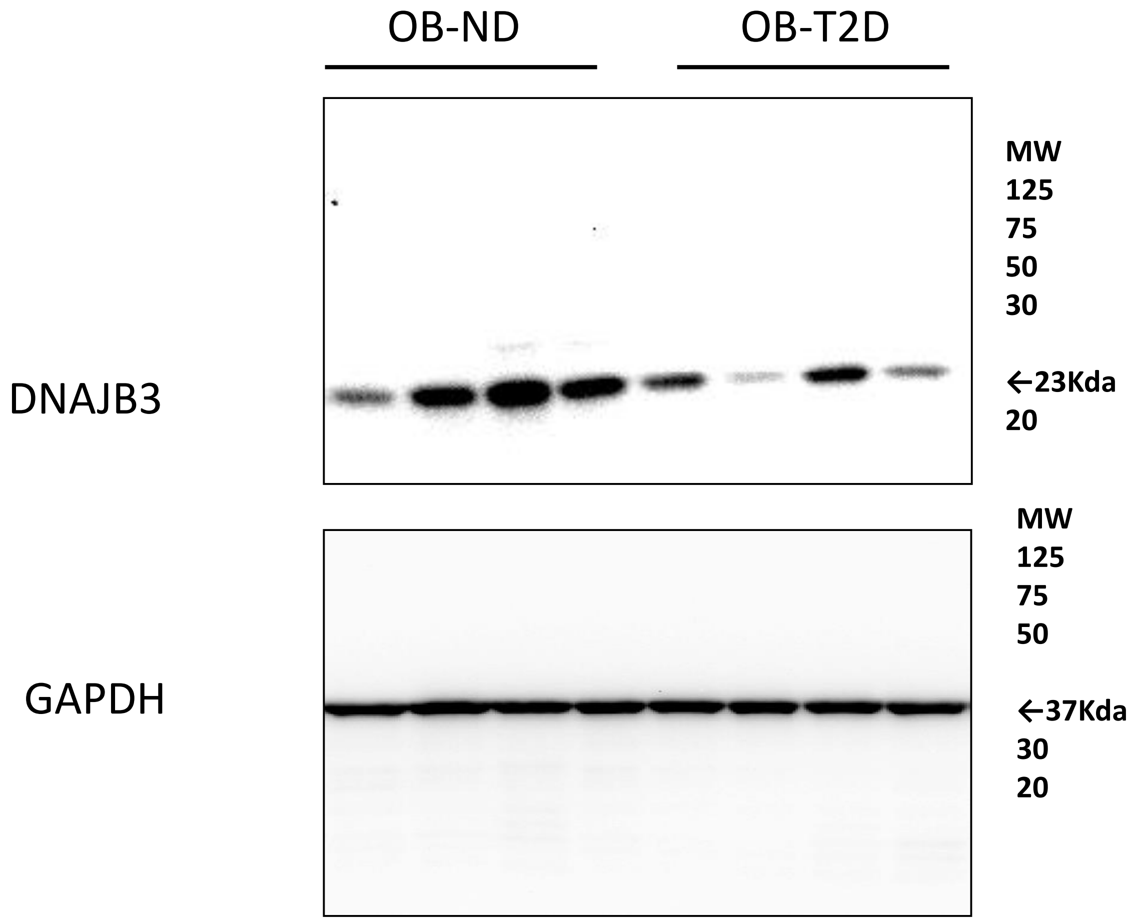

Figure 1

DNAJB3/HSP-40 cochaperone improves insulin signaling and enhances glucose uptake *in vitro* through JNK repression

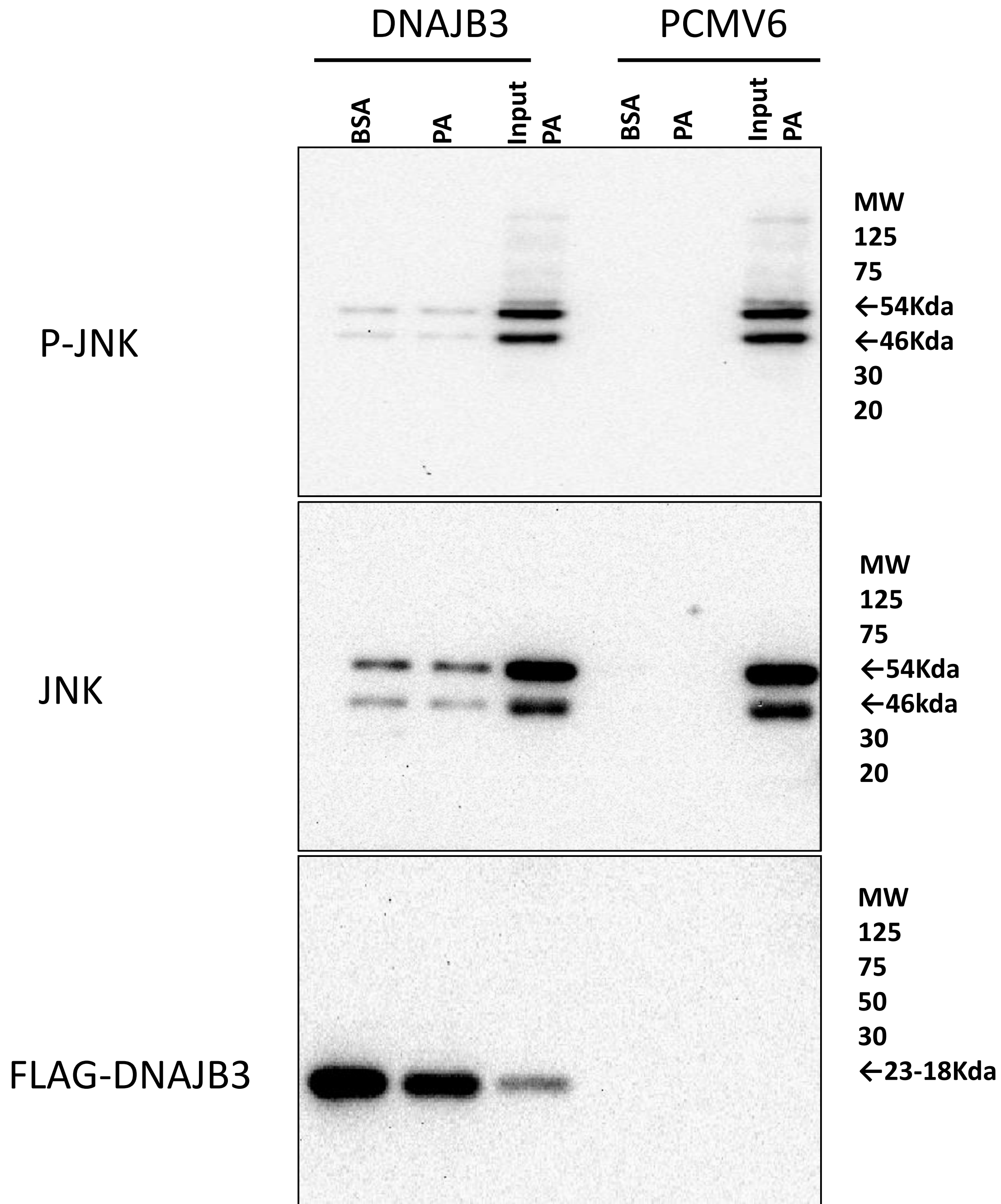

Figure 2A

DNAJB3/HSP-40 cochaperone improves insulin signaling and enhances glucose uptake *in vitro* through JNK repression

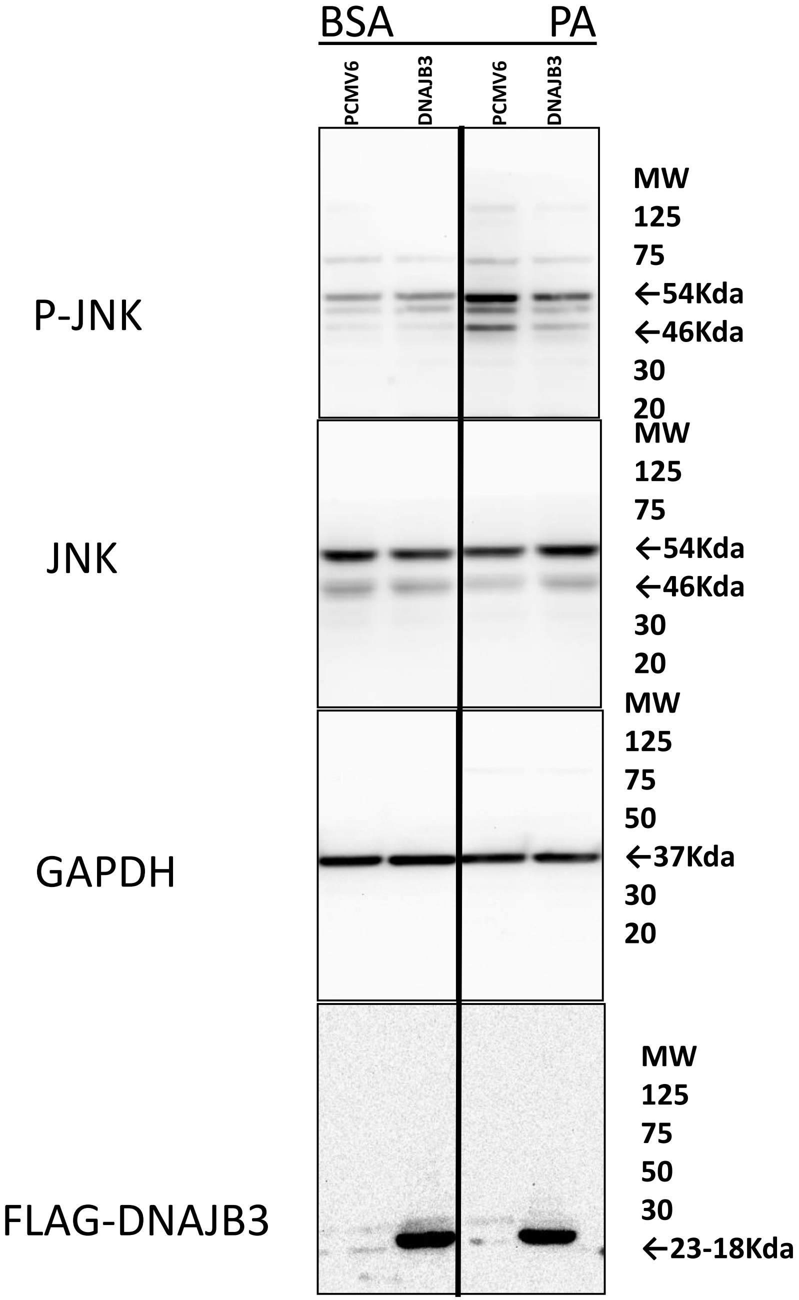

Figure 2B

DNAJB3/HSP-40 cochaperone improves insulin signaling and enhances glucose uptake *in vitro* through JNK repression

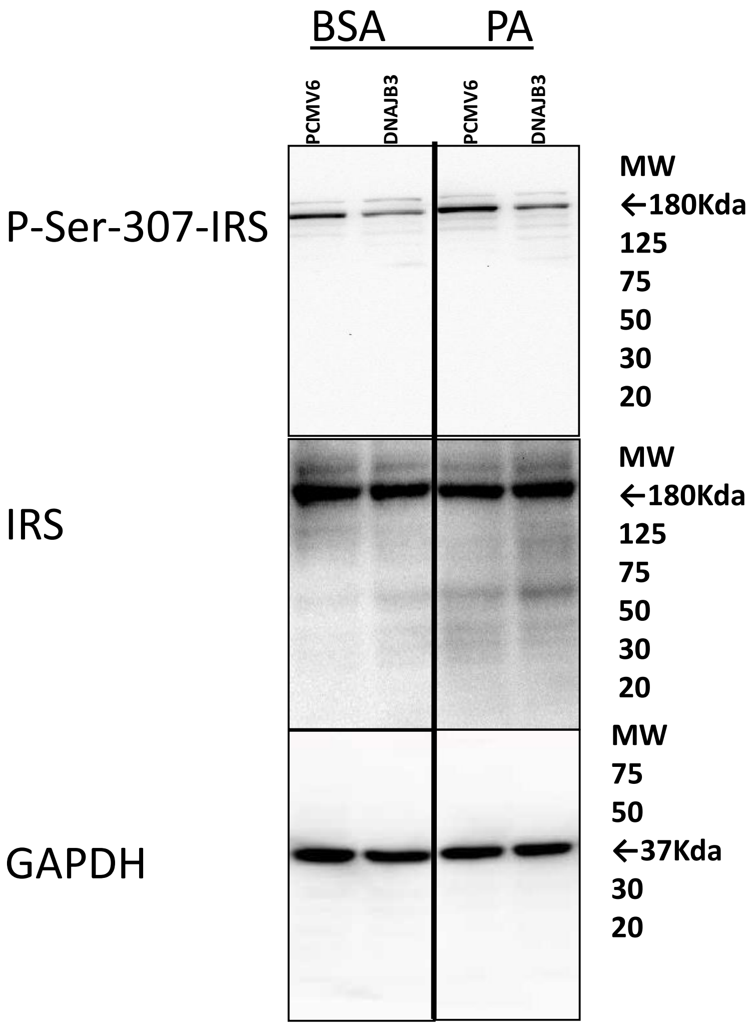

Figure 3A

DNAJB3/HSP-40 cochaperone improves insulin signaling and enhances glucose uptake *in vitro* through JNK repression

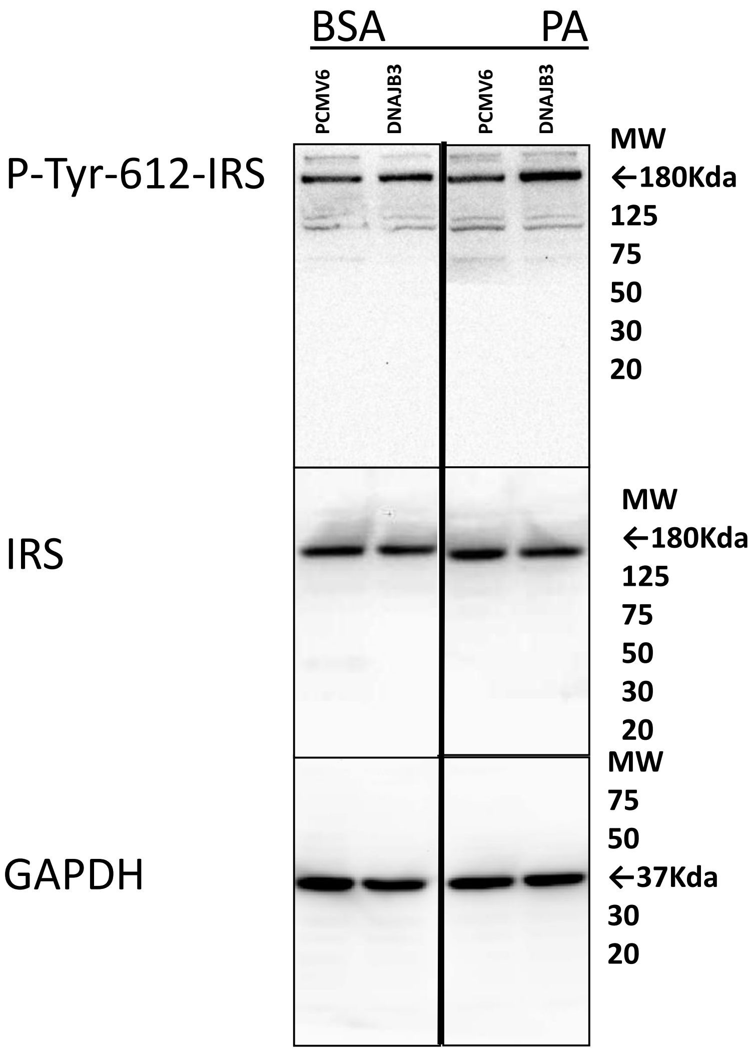

Figure 3B

DNAJB3/HSP-40 cochaperone improves insulin signaling and enhances glucose uptake *in vitro* through JNK repression

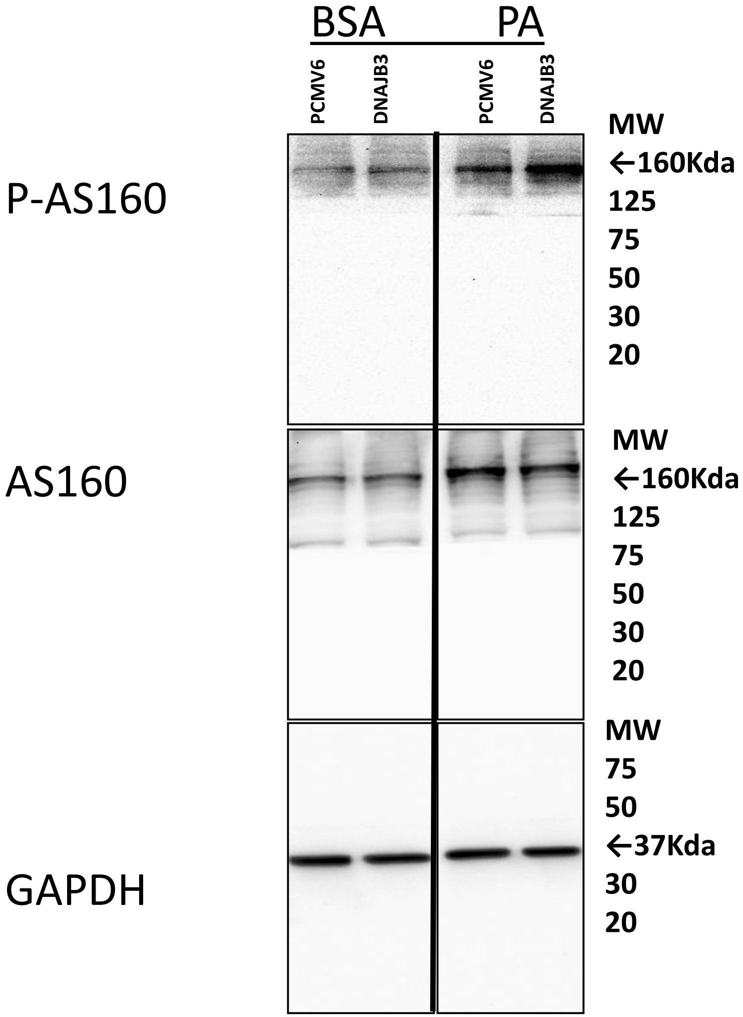

Figure 4A

DNAJB3/HSP-40 cochaperone improves insulin signaling and enhances glucose uptake *in vitro* through JNK repression

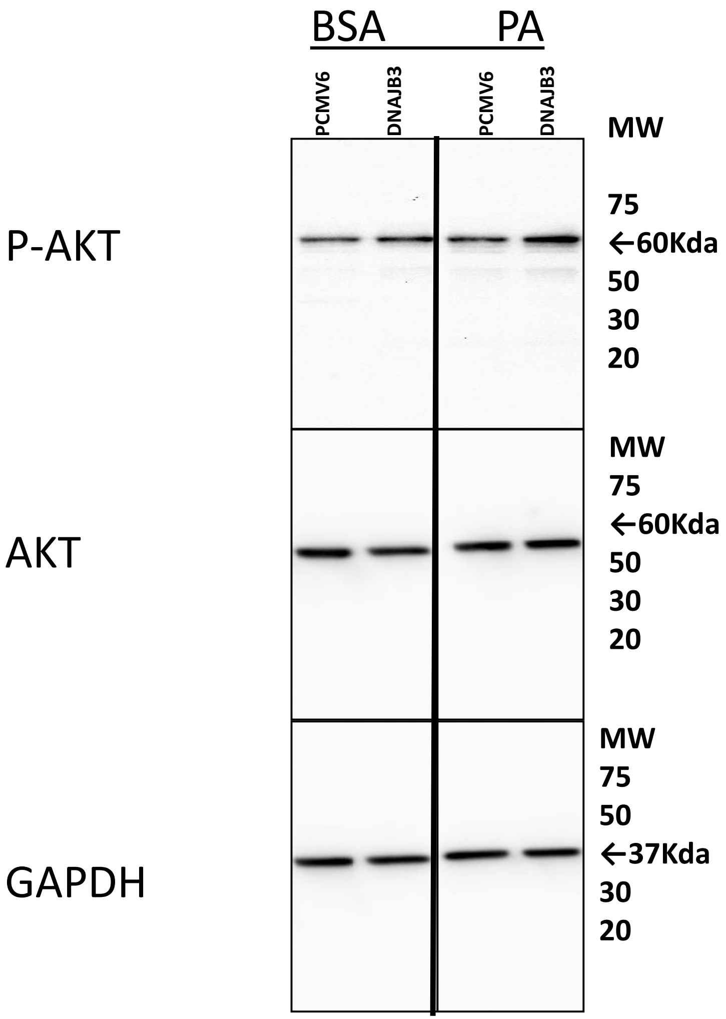

Figure 4B

DNAJB3/HSP-40 cochaperone improves insulin signaling and enhances glucose uptake *in vitro* through JNK repression

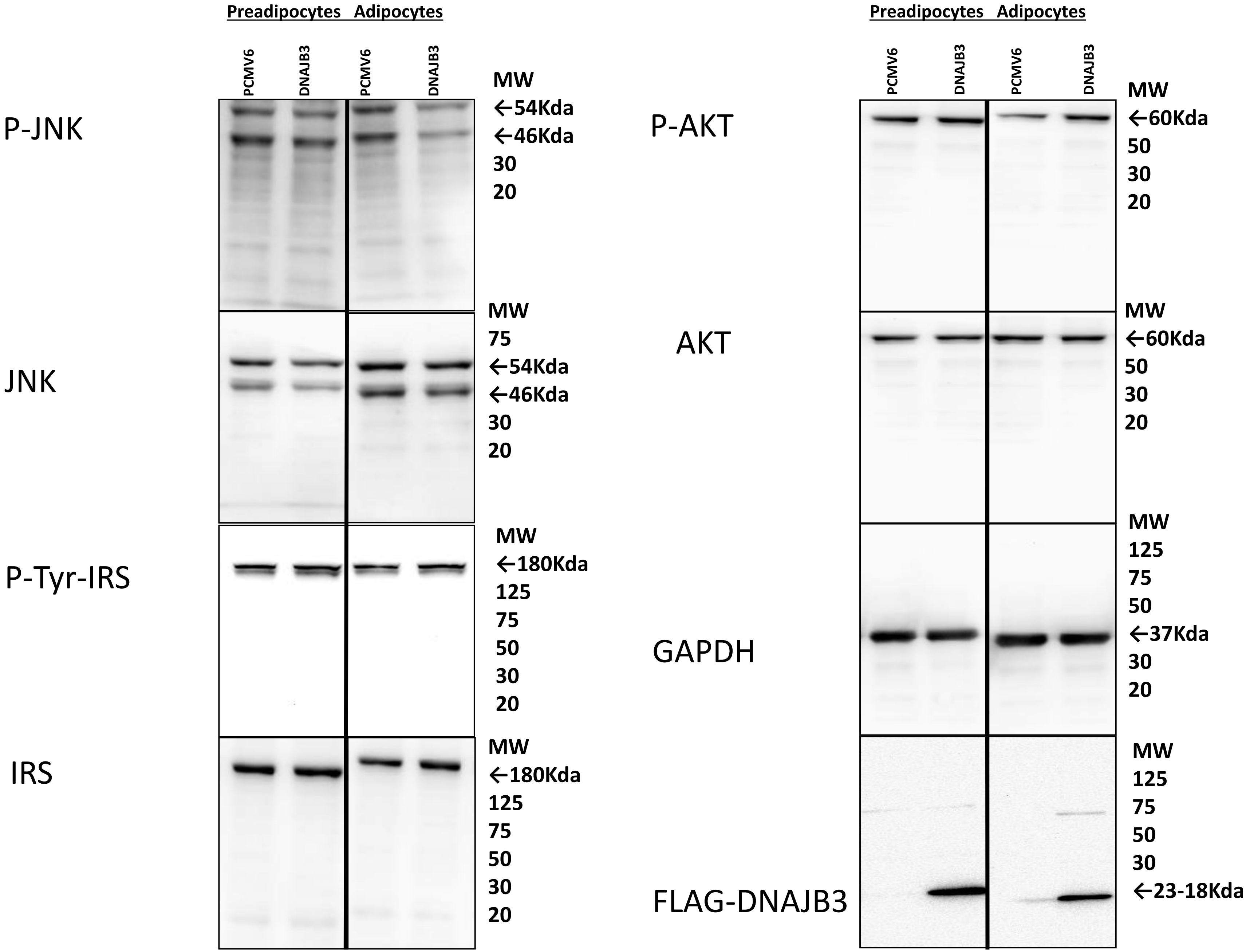

Figure 5
